# VP-net: an end-to-end deep learning network for elastic wave velocity prediction in human skin *in vivo* using optical coherence elastography

**DOI:** 10.3389/fbioe.2024.1465823

**Published:** 2024-10-14

**Authors:** Yilong Zhang, Jinpeng Liao, Zhengshuyi Feng, Wenyue Yang, Alessandro Perelli, Zhiqiong Wang, Chunhui Li, Zhihong Huang

**Affiliations:** ^1^ Centre of Medical Engineering and Technology, University of Dundee, Dundee, United Kingdom; ^2^ School of Physics and Engineering Technology, University of York, York, United Kingdom; ^3^ College of Medicine and Biological Information Engineering, Northeastern University, Shenyang, China

**Keywords:** optical coherence elastography, deep learning, convolutional neuronal network (CNN), surface acoustic wave (SAW), agar-based tissue-mimicking phantoms, *In vivo* human skin, closed comedones

## Abstract

**Introduction:**

Acne vulgaris, one of the most common skin conditions, affects up to 85% of late adolescents, currently no universally accepted assessment system. The biomechanical properties of skin provide valuable information for the assessment and management of skin conditions. Wave-based optical coherence elastography (OCE) quantitatively assesses these properties of tissues by analyzing induced elastic wave velocities. However, velocity estimation methods require significant expertise and lengthy image processing times, limiting the clinical translation of OCE technology. Recent advances in machine learning offer promising solutions to simplify velocity estimation process.

**Methods:**

In this study, we proposed a novel end-to-end deep-learning model, named velocity prediction network (VP-Net), aiming to accurately predict elastic wave velocity from raw OCE data of in vivo healthy and abnormal human skin. A total of 16,424 raw phase slices from 1% to 5% agar-based tissue-mimicking phantoms, 28,270 slices from in vivo human skin sites including the palm, forearm, back of the hand from 16 participants, and 580 slices of facial closed comedones were acquired to train, validate, and test VP-Net.

**Results:**

VP-Net demonstrated highly accurate velocity prediction performance compared to other deep-learning-based methods, as evidenced by small evaluation metrics. Furthermore, VP-Net exhibited low model complexity and parameter requirements, enabling end-to-end velocity prediction from a single raw phase slice in 1.32 ms, enhancing processing speed by a factor of ∼100 compared to a conventional wave velocity estimation method. Additionally, we employed gradient-weighted class activation maps to showcase VP-Net’s proficiency in discerning wave propagation patterns from raw phase slices. VP-Net predicted wave velocities that were consistent with the ground truth velocities in agar phantom, two age groups (20s and 30s) of multiple human skin sites and closed comedones datasets.

**Discussion:**

This study indicates that VP-Net could rapidly and accurately predict elastic wave velocities related to biomechanical properties of *in vivo* healthy and abnormal skin, offering potential clinical applications in characterizing skin aging, as well as assessing and managing the treatment of acne vulgaris.

## 1 Introduction

Skin, as the body’s largest organ, serves to regulate body fluid and temperature and forms a protective barrier shielding the organism against pathogens and injuries from the environment (Proksch et al., 2008). Skin disease is one of the most common human illnesses, affecting nearly 900 million people, more than one-third of the global population (Hay et al., 2014). Among these, acne vulgaris is a prevalent chronic skin inflammatory disease affecting up to 85% of late adolescents (Lynn et al., 2016), resulting in various consequences, including scarring, dyspigmentation, and psychological impacts (Ogé et al., 2019). However, there is currently no universally accepted assessment system for acne vulgaris.

The biomechanical properties of skin are primarily determined by its structural components (Joodaki and Panzer, 2018). Elastography is the functional modality to provide information on the biomechanical properties of tissues. Among different elastography modalities, optical coherence elastography (OCE), derived from optical coherence tomography (OCT), has an ultra-fast sampling rate, micrometer imaging resolutions and millimeter depth penetration (∼one to two mm) (Larin and Sampson, 2017). A notable branch of OCE technology is wave-based OCE, an *in situ* non-destructive approach that quantitatively estimates biomechanical properties in soft tissues using elastic waves (Liang and Boppart, 2009). Biomechanical properties, especially elasticity (Everett and Sommers, 2013), have been proven to be a potential biomarker for characterizing skin aging (Couturaud et al., 1995), understanding physiology, pathological cases, and monitoring treatment (Balbir-Gurman et al., 2002; Neto et al., 2013; Killaars et al., 2015). In OCE, wave propagation in tissue occurs when an elastic wave is generated by excitation and then transmits through other regions of the tissue. The velocity of the wave is intrinsically related to the biomechanical properties of the tissues (Kirby et al., 2017). OCE’s millimeter penetration depth confines motion measurements to regions near tissue boundaries, where surface acoustic waves (SAWs) are the dominant wave type (Zvietcovich and Larin, 2022). SAW velocities can be estimated by analyzing the phase term of the complex OCT signal. Typically, the phase difference between successive scans is utilized to detect sub-resolution axial differential displacement within a sample (Song et al., 2013), followed by the use of a time-of-flight approach to measure SAW velocities. By selecting an appropriate elasticity model, the biomechanical properties of the tissue can then be determined (Zvietcovich and Larin, 2022). While wave-based OCE has gained increasing interest in recent years, its application to *in vivo* skin conditions remains in its early stages. Two pre-clinical studies have shown the ability of wave-based OCE to characterize mechanical properties in animal models of systemic sclerosis (Du et al., 2016) and skin burns (Liu et al., 2024). However, only one wave-based OCE system has been translated to a clinical trial in human subjects for the assessment of systemic sclerosis *in vivo* (Liu et al., 2019). The major challenges limiting the clinical translation of OCE technology are the high level of expertise required and the inability to produce real-time results (Sun et al., 2011). In particular, biomechanical property analysis often demands complex image processing for wave feature extraction and velocity estimation (Song et al., 2015; Kirby et al., 2019), which could extend processing times to potentially several minutes or longer, limiting its use in real-time clinical settings.

Deep learning holds considerable promise for enhancing the efficiency of the processing of wave-based OCE by discerning and analyzing raw data. Currently, deep learning-assisted OCE analysis is still in the early stages. Schlaefer’s group (Neidhardt et al., 2020; Neidhardt et al., 2021; Neidhardt et al., 2023) demonstrated elastic velocity prediction for OCE data by using convolutional neural networks (CNNs) with dense connections. These methods have been proved based on homogeneous tissue-mimicking materials (Neidhardt et al., 2020; Neidhardt et al., 2021) and *ex vivo* chicken heart (Neidhardt et al., 2023) studies. However, there are inherent differences in structural (Labroo et al., 2021) and physical (Godin and Touitou, 2007) properties between heterogeneous animal and human tissue. Additionally, involuntary movements (Kirkpatrick et al., 2006) and breathing motion artefacts (Fang et al., 2019) frequently occur during *in vivo* human OCE acquisitions. Consequently, their CNN models might need to adapt the intricate textures of wave patterns from *in vivo* human data, instead of focusing on velocity prediction, leading to less optimal for *in vivo* human applications.

In this study, we propose a novel velocity prediction network (VP-Net), that predicts bulk (body) wave velocities in *vivo* human healthy and abnormal skin sites from raw OCE data. The network architecture incorporates a squeeze-and-excitation (SE) block (Hu et al., 2018) and a separable convolution block, enabling efficient feature reuse and integration without significantly increasing model complexity. Compared to existing CNN models, VP-Net could accurately predict elastic wave velocity from each raw phase slice directly, maintaining the lowest model complexity and inference time. VP-Net demonstrated high accuracy in predicting elastic wave velocities in multiple healthy skin sites and distinguishing age-related velocity changes between 20s and 30s age groups. Closed comedones, a type of acne lesions (Lavers, 2014), were also investigated in this study. VP-Net’s successfully predicted high velocities in comedones, indicating elevated skin elasticity. To the best of our knowledge, this is the first study to quantify the biomechanical properties of facial acne lesions using OCE technology and to develop an elastic wave velocity prediction model in human *in vivo* using deep learning. VP-Net achieved a processing speed of 1.32 ms per slice, approximately 100 times faster than a conventional velocity estimation method. Therefore, VP-Net offers real-time elastic wave velocity prediction in human skin *in vivo*, providing potential clinical applications in characterizing skin aging, as well as assessing and managing the treatment of acne vulgaris.

Our study has five main contributions: 1) Our model demonstrated consistent and repeatable velocity predictions on tissue-mimicking phantoms, which are homogenous and have consistent biomechanical properties for each concentration. 2) To the best of our knowledge, this is the first study to deploy a deep learning method to directly predict biomechanical property-related velocities for *in vivo* human healthy and abnormal datasets, showcasing its potential for skin condition diagnosis. 3) We conducted a comprehensive comparison with various neural networks and an ablation study on VP-Net to validate the efficacy of our proposed model. 4) Compared to existing models, the proposed VP-Net has the fastest inference time and the lowest model complexity while providing accurate SAW velocity predictions, even when applications shifted from tissue-mimicking materials to *in vivo* human skin. 5) We used gradient-based class activation maps (Grad-CAM) to visualize the model’s process in predicting velocities.

This paper is structured as follows: The Methods section describes the details of our proposed velocity prediction deep learning model and the OCE data processing strategies to generate raw phase slices and ground truth velocities. The Results section presents the performance metrics, ablation study, and visual explanations of our network’s efficacy in predicting velocities. Additionally, the predicted bulk velocities of agar phantoms and healthy skin sites from participants across two age groups, as well as abnormal skin, are shown. Finally, we conclude the paper with a summary of our key contributions, a discussion on the factors affecting model performance, and potential improvements for future research.

## 2 Methods

### 2.1 Definition of deep learning-based OCE velocity prediction pipeline

**FIGURE 1:**
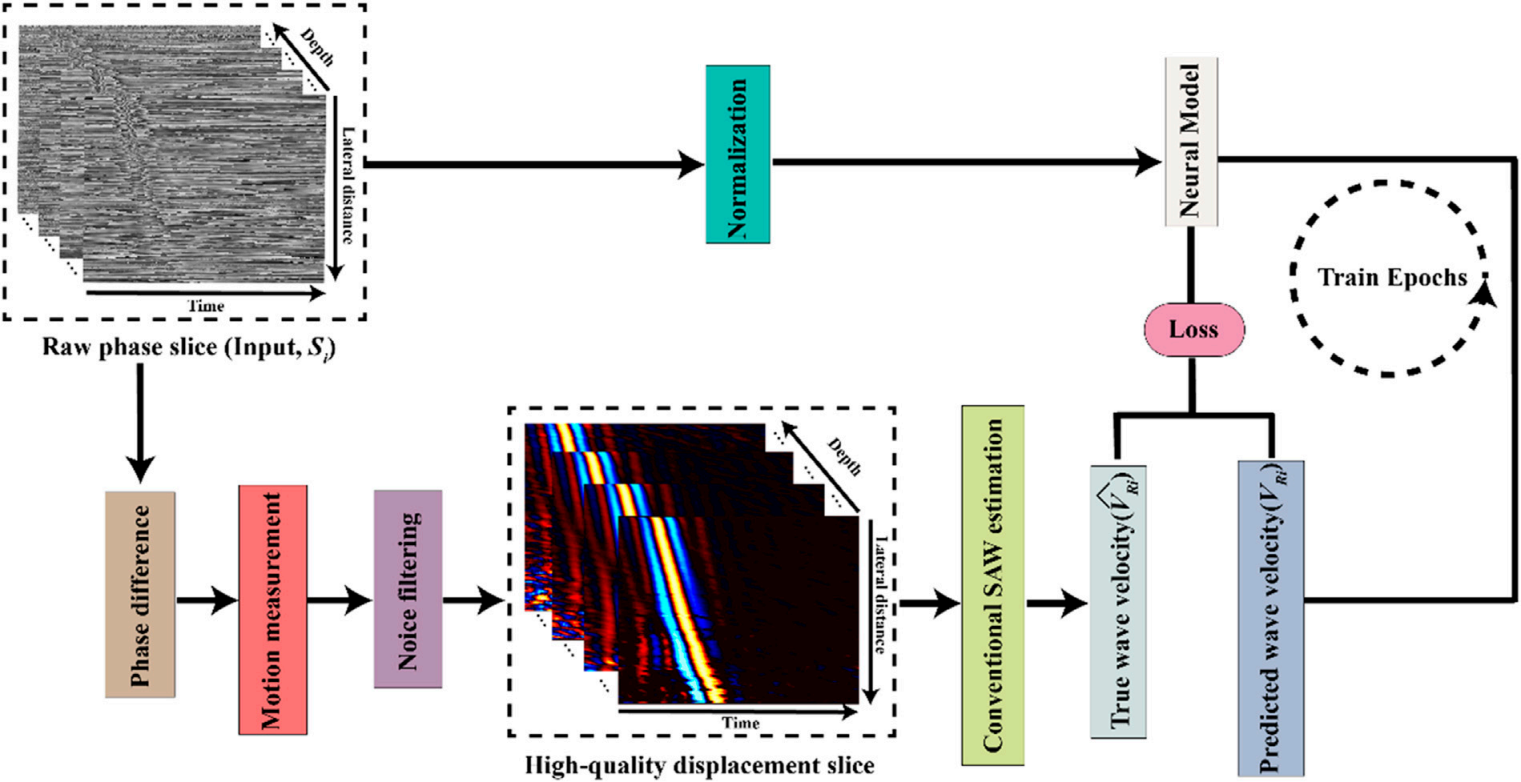
Schematic of the deep learning-based optical coherence tomography elastography (OCE) velocity prediction pipeline.

To facilitate accurate and fast determination of biomechanical properties, specifically Young’s modulus, from OCE imaging, an automated prediction of bulk SAW velocity is essential. Figure 1 illustrates a schematic of our proposed OCE velocity prediction pipeline.

In this study, we designed our neural model to function as a linear regression model to predict SAW velocity from the input of single raw phase slices, $S_{i}\in R_{+}^{M\times M}$ , with $i=1,\cdots ,L\;\left(L\leq 300\right)$ where the number of images *L* is specified according to the dataset used in the experimental results and M=320 . The definition is provided in Equation 1:

$$\hat{V_{Ri}}=f\left(S_{i}\right)$$ (1)

where $\hat{V_{Ri}}\in R_{+},\text{with }i=1,\cdots ,L$ denotes the model-predicted velocity at a given depth layer $\left(i\right)$ , and f is the neural model employed in our study. During the training stage, the model-predicted velocity was compared with the ground truth velocity $\left(V_{Ri}\right)$ calculated by a conventional elastic wave velocity estimation (Song et al., 2013). This comparison facilitated the calculation of the training loss, which subsequently guided the updating of the model’s trainable parameters.

### 2.2 Velocity prediction network (VP-Net) architecture

**FIGURE 2:**
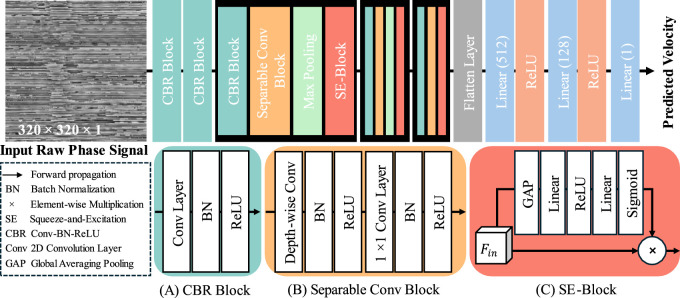
Architecture of VP-Net model. **(A)** CBR Block. **(B)** Separable Conv Block. **(C)** SE-Block.

The architecture of our proposed velocity prediction network (VP-Net) is depicted in Figure 2. VP-Net includes four downsample stages to extract the features and reduce the size of the feature maps from the input 2D raw phase signal in spatial-temporal dimensions, thereby predicting the velocity. VP-Net has fewer parameters and less computational demand than models like VGG16 (Simonyan and Zisserman, 2014) and ResNet18 (He et al., 2016), significantly reducing the resources for model inference and training. VP-Net is mainly formed with three blocks: convolution-batch normalization-ReLU (CBR) block, separable convolution block, and SE-Block. The network is described in detail in the following sections.

#### 2.2.1 CBR block

As shown in Figure 2A, the CBR Block consists of a 2D convolution layer (Conv2D), a batch normalization layer (BN), and a ReLU activation layer. Taking the input is $F_{\text{in}}$ , and the output is $F_{\text{out}}$ , the forward process of the CBR block can be written as Equation 2:

$$F_{\text{out}}=\text{ReLU}\left(\text{BN}\left(\text{Conv}2D\left(F_{\text{in}}\right)\right)\right)$$ (2)

In terms of the setting of five CBR blocks in VP-Net, as shown in Figure 2, the first CBR block has a kernel size of 11, a stride of 4, and a filter size of 16, providing a trainable and overlapped image patch extraction function. Moreover, the large kernel size (i.e., 11) can provide a larger receptive field, which is essential to this study since the input raw phase signals include a time signal. The second CBR block has a kernel size of 3, a stride of 1, and a filter size of 16, further extracting the features from the image patches from the first CBR block. The third and fourth CBR blocks have the same kernel size of 7, a stride of 2, and a filter size of 32 and 64, respectively. The fifth CBR block has a kernel size of 3, a stride of 1, and a filter size of 128.

#### 2.2.2 Separable conv block

To achieve a lower model complexity, we introduced the separable convolution block to VP-Net. Compared to the 2D convolution layer, a separable convolution block can extract the features based on the channel-wise and spatial-wise, while reducing the model complexity and computational resource demanded. Assume the input feature is $F_{\text{in}}$ , and the output feature is $F_{\text{out}}$ , the forward process of the separable conv block can be written as Equation 3:

$$F_{\text{out}}=\text{ReLU}\left(\text{BN}\left({\text{Conv}}_{1\times 1}\left(\text{ReLU}\left(\text{BN}\left({\text{Conv}}_{\text{Dw}}\left(F_{\text{in}}\right)\right)\right)\right)\right)\right)$$ (3)

Regarding the setup of the three separable conv blocks in VP-Net, all depth-wise convolution layers and 1 × 1 convolution layers have the same filter size as the $F_{\text{in}}$ . The kernel size of all depth-wise convolution layers is 3. The stride of all depth-wise convolution layers and 1 × 1 convolution layers is 1.

#### 2.2.3 SE block

To improve the efficiency of the feature reuse, we also introduced the squeeze-and-excitation (SE) block (Hu et al., 2018) to VP-Net, which can improve model performance by adaptively recalibrating channel-wise feature responses, thereby improving the model’s representational power and accuracy of velocity prediction. Taking the input as $F_{\text{in}}$ with a shape of H × W × C, and the output is $F_{\text{out}}$ , the forward process of the SE block can be expressed as Equation 4:

$$F_{\text{out}}=F_{\text{in}}\times \text{Sigmoid}\left({\text{Linear}}_{1}\left(\text{ReLU}\left({\text{Linear}}_{2}\left(\text{GAP}\left(F_{\text{in}}\right)\right)\right)\right)\right)$$ (4)

where GAP is global averaging pooling. After processing by GAP, the shape of the feature is converted from H × W × C to 1 × 1 × C. Linear stands for linear projection operation and the units of the ${\text{Linear}}_{1}$ is set as C/4, and the units of the ${\text{Linear}}_{2}$ is set as C.

### 2.3 Data pre-processing

The acquired raw OCE volume (512 depth × 512 lateral × 512 time pixels) were cropped to 320 × 320 pixels along the lateral and time axes to get rid of the head of the piezoelectric actuator and retain the region of interest. The raw phase $\left(\varphi \left(x,z,t\right)\right)$ of the complex OCT data was linear normalized to be in the range of 0–1 by Equation 5:

$$\varphi_{Nor}\left(x,z,t\right)=\left(\frac{\varphi \left(x,z,t\right)}{\pi }\times 0.5\right)+0.5$$ (5)

The temporal-spatial normalized raw phase slices served as the input of deep learning models.

### 2.4 Ground truth elastic wave velocity estimation

In order to provide accurate bulk SAW velocities as ground truth for model development, a conventional wave velocity estimation method was employed, including phase change measurement, noise filter applications for wave extraction and a time-of-flight approach for velocity estimation. First, the phase difference (Δ*φ*(*x, z, t*)) between two consecutive A-lines (along the temporal axis) at each spatial position was calculated to compute deformation. The axial displacement at each lateral location was then measured from the phase difference (Wang et al., 2007). Next, the following noise filters were applied to the spatial-temporal displacement data. A directional filter was applied to minimize the distortion effect by reflected/refracted elastic waves on the original forwarding waves (Kirby et al., 2019). A low pass filter with a cutoff frequency of 2 kHz was applied to further eliminate high-frequency noise (Kirby et al., 2019). The remaining noise was reduced by using a 3D median filter of the kernel size of 11 × 5 in all directions (Neidhardt et al., 2020). Finally, the displacement was normalized by dividing it by the maximum value of each particle along the time axis. For velocity estimation, a time-of-flight approach (Song et al., 2013) was used, which involved tracking the main peak of the waveform along the propagation direction. In this work, the main peak of the wavefront is defined as the maximum of the normalized displacement along lateral locations. For a given depth layer ( i ), the ground truth bulk velocity ( $V_{Ri}$ ) was estimated by calculating the slope of the space-time main wavefront peak curve along lateral locations, expressed as Equation 6 (Song et al., 2013):

$$V_{Ri}=\frac{\Delta x}{\Delta t}$$ (6)

Where $V_{Ri}\in R_{+},\text{with }i=1,\cdots ,L$ , and Δx represents the distance traveled by the main peak of the SAW wavefront along lateral locations during time shift Δt . SAW velocity over the depth layer ( i ) was obtained using linear least squares regression fitting the time shits to the corresponding propagation distances (Lan et al., 2021), continuing until L=300 or until reaching the maximum iteration limit when the relative difference of two continuous coefficient estimates exceeded 1 × 10^-6^ $\left(L<300\right).$ For the abnormal skin dataset, only the lesion region was selected and fitted. In this study, the above procedures for SAW velocity estimation were designed to provide accurate ground truth for generating the labels needed during supervised training and did not influence the model’s performance in velocity prediction once trained.

### 2.5 Experimental data acquisition and dataset

#### 2.5.1 Agar-based tissue-mimicking phantom

Eight concentrations of agar-based tissue-mimicking phantoms ranging from 1% to 5% with an interval of 0.5% were fabricated. The general protocol for producing the agar phantom has been described in detail in our previous study (Li et al., 2015). Each phantom underwent scanning at three locations with three repetitions. For algorithm development, 16,424 normalized raw phase slices of agar phantoms (sourced from 7 OCE scans for each concentration) were used for model training. A random selection of 1,147 slices was used for model validation, and 4,854 slices (sourced from 2 OCE scans for each concentration) were used for model testing.

#### 2.5.2 *In vivo* human healthy skin

Sixteen healthy adults, including nine males and seven females from the 20s and 30s age groups, with no history of skin or medical conditions, were enrolled in this study. Each participant underwent scanning at three sites (palm, forearm, and back of hand) with three acquisitions at each site. The study was approved by the School of Science and Engineering Research Ethics Committee (SSEREC) of the University of Dundee, which also conformed to the tenets of the Declaration of Helsinki. Informed consent was obtained from each subject prior to the OCE imaging.

For algorithm development, overall, 28,270 normalized raw phase slices were produced from 16 participants’ OCE data. Of them, 17,671 slices (sourced from 10 participants, with an equal split of 5 each from the 20s and 30s age groups) were used for model training, 4,340 slices from 2 participants (one from each age group) were set aside for validation, and 6,259 slices from 4 independent participants (two from each age group) were used for model test preventing data leakage.

#### 2.5.3 *In vivo* human abnormal skin

Seven facial closed comedones from two enrolled adults were scanned using OCE imaging, with three acquisitions taken for each comedo. For model training, we utilized 580 raw phase slices sourced from 3 OCE scans. An additional 129 slices from 1 OCE scan were used for validation, and 641 slices from 3 OCE scans were used for testing.

The velocities of agar phantoms have been well studied (Yang et al., 2022; Brewin et al., 2015), and the wave patterns of homogeneous agar phantoms tend to be straightforward and clear (Wang and Larin, 2015). Thus, the existing agar phantom datasets served as a validation of our VP-Net’s accuracy. Importantly, the wide range of agar phantom velocities covered both healthy and abnormal human skin velocities, thereby enhancing the model’s performance through convergence of predictions. The imbalance between the smaller number of agar phantom slices and the larger number of human skin slices ensured that the model placed more weight on learning from *in vivo* data, characterized by multiple wave patterns, high noise and artifacts.

### 2.6 Experimental setup and data acquisition

**FIGURE 3:**
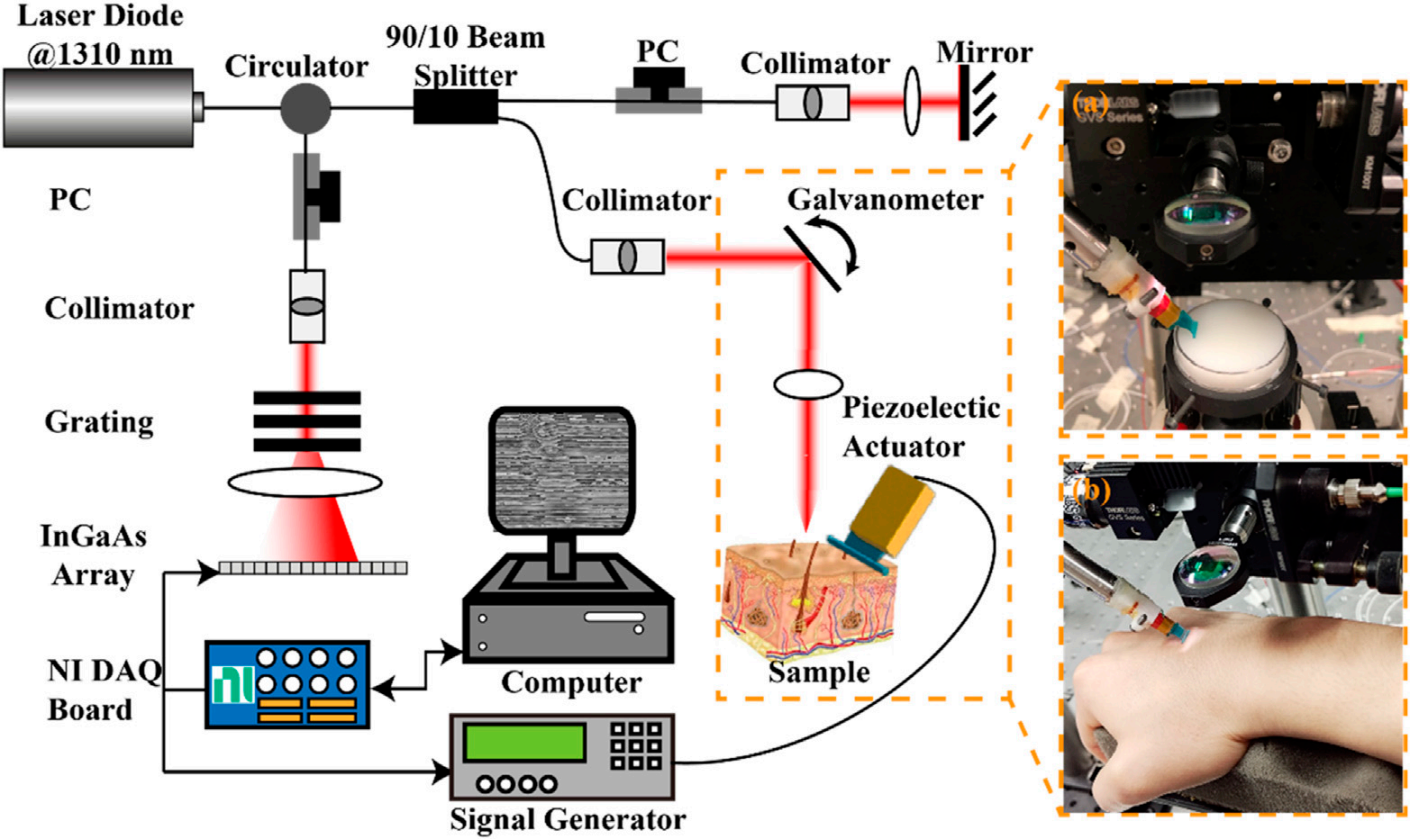
Schematic of the experimental setup for the generation and detection of SAW on sample using a piezoelectric actuator and the PhS-OCT system, and photographs of **(A)** agar-based tissue-mimicking phantom and **(B)** *in vivo* human skin data acquisition. DAQ, Data acquisition; NI, national instrument; PC: polarization controller; PhS-OCT, phase-sensitive optical coherence tomography.

A lab-built OCE system consisting of a phase-sensitive OCT (PhS-OCT) system and an external SAW generation system was used in this study. Figure 3 presents the schematic of the experimental set of the OCE system, along with photographs capturing the agar-based tissue-mimicking phantom (Figure 3, a) and *in vivo* human skin (Figure 3B) during data acquisition. The PhS-OCT, with a central wavelength of 1,310 ± 110 nm and sampling frequency of 92 kHz, detected mechanically induced SAWs in the skin. The axial sampling distance and lateral sampling distance were measured as 4.7 μm/pixel and 21.7 μm/pixel, respectively.

A piezoelectric actuator (PC4QR, Thorlabs Inc., Newton, NJ, United States of America) was set at an angle of 45° contact with the skin to generate SAW. The piezoelectric actuator was triggered by the waveform generator, which could generate the square wave with a frequency of 2 kHz, a peak-to-peak voltage of 10 mV, and a duty cycle of 60%.

An M-B scanning protocol was employed to acquire the propagation of the SAWs. One complete acquisition was completed within 3.9 s. The size of the effective imaging plane was ∼2 mm × 11 mm (depth × lateral distance). All data was acquired through a customized LabVIEW interface (LabView 2020; National Instruments, Austin, TX, United States) and stored in the computer for processing.

### 2.7 Model training details

All neural networks used in the study were built and trained based on TensorFlow 2.9.0 backend (Abadi et al., 2016). The training took place on a Nvidia RTX 4090 with 24 GB memory. The training epoch of VP-Net was set as 1,000, with a batch size of 32. An Adam optimizer (Kingma and Ba, 2014) with a learning rate of 0.001 was used to update trainable weights in the models. The mean-absolute-error (MAE) was utilized as the loss function since we found that the mean-square-error (MSE) function would bring the unstable training of all neural networks in this study. An early stop strategy was used to save the best performance model’s weights when the metrics validation loss of MAE was not decreased in 30 training epochs, preventing overfitting during the model training. Data augmentation, such as rotation and flipping, were not used since those methods would affect the patterns and properties of perturbations, leading to unstable training.

### 2.8 Evaluation metrics

To evaluate the performance of the proposed deep learning-based velocity prediction for OCE, MSE and MAE were used to calculate the difference between the model-predicted velocity ( $\hat{V_{Ri}}$ ) and the ground truth velocity ( $V_{Ri}$ ) obtained by the linear fitting of the wavefront curve. The MSE and MAE are given by Equations 7, 8, respectively.

$$\text{MSE}\left(V_{Ri},\hat{V_{Ri}}\right)={\left(V_{Ri}-\hat{V_{Ri}}\right)}^{2}$$ (7)

$$\text{MAE}\left(V_{Ri},\hat{V_{Ri}}\right)=\left|V_{Ri}-\hat{V_{Ri}}\right|$$ (8)

## 3 Results

### 3.1 Comparison with neural networks on velocity prediction

The performance of our VP-Net on bulk SAW velocities of agar and human skin datasets was evaluated with various published deep-learning networks, including the VGG16/19 (Simonyan and Zisserman, 2014), ResNet18/34/50/101 (He et al., 2016), DenseNet121/169 (Huang et al., 2017), and MobileNetV2 (Sandler et al., 2018). The training details and training strategy of the compared-used models were consistent with VP-Net. The evaluation was based on the test set to avoid data leakage. The evaluation metrics were MAE and MSE, and a lower value of resultant indicated a more accurate velocity prediction.

Table 1 and Table 2 demonstrate the comparison results of MAE and MSE among various networks based on the eight concentrations of the agar-based tissue-mimicking phantoms from the test set. VP-Net had the best MSE and MAE performance in the 1.5%, 3.0% and 4.0% agar phantoms. Furthermore, VP-Net had similar MAE (0.225) and MSE (0.393) values to the mobileNetV2 (MAE: 0.183; MSE: 0.325) in the 2.5% agar phantom. However, VP-Net had a relatively low performance in 1.0%, 3.5%, and 5.0% agar phantoms from the test set.

**TABLE 1:** MSE of neural networks for SAW velocity prediction on agar phantoms.

Model	1.0% agar	1.5% agar	2.0% agar	2.5% agar	3.0% agar	3.5% agar	4.0% agar	4.5% agar	5.0% agar
VGG16	1.517 ± 0.309	0.223 ± 0.236	0.200 ± 0.237	0.241 ± 0.255	0.069 ± 0.099	0.760 ± 0.963	0.181 ± 0.226	0.468 ± 0.595	0.797 ± 1.007
VGG19	1.519 ± 0.366	0.213 ± 0.226	0.167 ± 0.197	0.246 ± 0.258	0.077 ± 0.108	0.725 ± 1.020	0.188 ± 0.286	0.280 ± 0.377	**0.513 ± 0.648**
ResNet18	4.799 ± 3.310	0.290 ± 0.365	0.330 ± 0.784	0.367 ± 0.432	0.849 ± 0.474	0.634 ± 0.776	2.354 ± 1.342	3.406 ± 2.068	5.169 ± 3.287
ResNet34	0.830 ± 0.440	0.205 ± 0.225	0.208 ± 0.330	0.203 ± 0.293	0.491 ± 0.287	0.565 ± 0.750	0.261 ± 0.305	0.525 ± 0.659	0.858 ± 1.137
ResNet50	**0.217 ± 2.547**	0.290 ± 0.345	1.500 ± 6.456	5.641 ± 4.618	1.746 ± 0.609	0.896 ± 1.154	0.267 ± 0.364	**0.184 ± 0.246**	2.334 ± 1.466
ResNet101	1.885 ± 0.329	0.305 ± 0.306	**0.152 ± 0.199**	0.294 ± 0.274	1.107 ± 0.383	1.571 ± 1.802	2.683 ± 1.255	3.081 ± 1.712	5.067 ± 2.817
DenseNet121	5.253 ± 2.301	1.094 ± 0.677	0.796 ± 0.925	0.388 ± 0.687	0.089 ± 0.103	**0.503 ± 0.625**	0.643 ± 0.577	1.264 ± 1.134	2.286 ± 1.883
DenseNet169	4.298 ± 2.212	0.559 ± 0.488	0.927 ± 1.126	0.247 ± 0.467	0.291 ± 0.226	0.558 ± 0.674	1.972 ± 1.071	2.682 ± 1.594	4.932 ± 2.783
MobileNetV2	5.024 ± 1.334	1.280 ± 0.786	0.495 ± 0.509	**0.183 ± 0.309**	0.162 ± 0.153	0.590 ± 0.788	0.266 ± 0.315	0.490 ± 0.606	1.058 ± 1.292
VP-Net	1.158 ± 1.294	**0.121 ± 0.128**	0.369 ± 0.420	0.225 ± 0.256	**0.057 ± 0.090**	0.742 ± 1.350	**0.149 ± 0.192**	0.636 ± 0.696	0.995 ± 1.074

The results shown as mean ± standard deviation; The best value of MSE, for each agar phantom highlighted in bold.

**TABLE 2:** MAE of neural networks for SAW velocity prediction on agar phantoms.

Model	1.0% agar	1.5% agar	2.0% agar	2.5% agar	3.0% agar	3.5% agar	4.0% agar	4.5% agar	5.0% agar
VGG16	1.225 ± 0.125	0.394 ± 0.260	0.364 ± 0.260	0.415 ± 0.262	0.209 ± 0.159	0.709 ± 0.507	0.348 ± 0.246	0.557 ± 0.397	0.727 ± 0.518
VGG19	1.224 ± 0.143	0.385 ± 0.254	0.332 ± 0.238	0.421 ± 0.263	0.223 ± 0.165	0.681 ± 0.511	0.336 ± 0.274	0.426 ± 0.315	**0.583 ± 0.417**
ResNet18	2.079 ± 0.689	0.436 ± 0.316	0.417 ± 0.395	0.507 ± 0.332	0.879 ± 0.274	0.652 ± 0.458	1.462 ± 0.464	1.750 ± 0.586	2.151 ± 0.735
ResNet34	0.881 ± 0.231	0.377 ± 0.250	0.361 ± 0.279	0.362 ± 0.267	0.666 ± 0.219	0.607 ± 0.444	0.424 ± 0.285	0.592 ± 0.418	0.747 ± 0.548
ResNet50	**0.258 ± 0.387**	0.444 ± 0.305	0.602 ± 1.067	2.248 ± 0.767	1.301 ± 0.230	0.766 ± 0.557	0.413 ± 0.311	**0.344 ± 0.25**6	1.440 ± 0.510
ResNet101	1.368 ± 0.121	0.466 ± 0.298	**0.322 ± 0.219**	0.470 ± 0.270	1.035 ± 0.190	1.040 ± 0.699	1.588 ± 0.402	1.681 ± 0.507	2.158 ± 0.641
DenseNet121	2.243 ± 0.472	0.988 ± 0.344	0.759 ± 0.470	0.464 ± 0.415	0.250 ± 0.165	**0.579 ± 0.410**	0.709 ± 0.375	0.998 ± 0.518	1.365 ± 0.651
DenseNet169	2.016 ± 0.485	0.659 ± 0.354	0.809 ± 0.522	0.367 ± 0.335	0.490 ± 0.224	0.613 ± 0.428	1.344 ± 0.408	1.557 ± 0.508	2.124 ± 0.649
MobileNetV2	2.223 ± 0.290	1.071 ± 0.365	0.599 ± 0.370	**0.325 ± 0.278**	0.353 ± 0.195	0.619 ± 0.455	0.427 ± 0.289	0.573 ± 0.402	0.843 ± 0.589
VP-Net	0.913 ± 0.569	**0.295 ± 0.185**	0.505 ± 0.338	0.393 ± 0.266	**0.184 ± 0.150**	0.672 ± 0.539	**0.313 ± 0.227**	0.673 ± 0.428	0.837 ± 0.542

The results shown as mean ± standard deviation; The best value of MAE, for each agar phantom highlighted in bold.

Table 3 shows the comparison of VP-Net with various networks based on *in vivo* human healthy and abnormal skin datasets. The proposed VP-Net performed the best for the back of hand (MSE: 1.585; MAE: 0.992) and forearm (MAE: 0.997). The ResNet101 demonstrated the lowest MSE (1.844) and MAE (1.133) for the palm. For the closed comedones dataset, VP-Net showed the second-best performance, with MSE of 1.051 and MAE of 0.863.

**TABLE 3:** MSE and MAE of neural networks for SAW velocity prediction on *in vivo* human skin.

Model	Back of hand		Palm		Forearm		Closed comedones	
	MSE	MAE	MSE	MAE	MSE	MAE	MSE	MAE
VGG16	1.805 ± 2.016	1.130 ± 0.727	2.264 ± 2.664	1.228 ± 0.870	2.054 ± 2.574	1.027 ± 0.834	**0.643 ± 0.665**	**0.702 ± 0.388**
VGG19	2.373 ± 2.998	1.287 ± 0.847	2.428 ± 2.850	1.269 ± 0.904	**1.725 ± 2.592**	1.012 ± 0.837	1.118 ± 1.048	0.929 ± 0.506
ResNet18	21.424 ± 13.997	4.274 ± 1.777	4.461 ± 4.919	1.759 ± 1.169	10.404 ± 8.477	2.890 ± 1.432	4.045 ± 2.673	1.856 ± 0.774
ResNet34	10.797 ± 10.183	2.893 ± 1.558	4.739 ± 5.169	1.820 ± 1.195	5.080 ± 6.163	1.866 ± 1.264	5.481 ± 3.232	2.206 ± 0.783
ResNet50	3.340 ± 7.083	1.378 ± 1.200	7.981 ± 8.224	2.405 ± 1.482	4.507 ± 9.203	1.478 ± 1.524	12.435 ± 7.287	3.363 ± 1.059
ResNet101	1.941 ± 2.484	1.143 ± 0.796	**1.844 ± 2.105**	**1.133 ± 0.748**	2.052 ± 3.080	1.103 ± 0.914	1.474 ± 1.505	1.050 ± 0.609
DenseNet121	11.885 ± 8.404	3.170 ± 1.355	3.342 ± 3.857	1.502 ± 1.042	7.578 ± 6.893	2.448 ± 1.260	3.352 ± 2.467	1.661 ± 0.771
DenseNet169	7.913 ± 6.914	2.506 ± 1.279	3.281 ± 3.914	1.489 ± 1.032	5.470 ± 5.626	2.033 ± 1.157	2.714 ± 2.436	1.451 ± 0.781
MobileNetV2	10.498 ± 7.765	2.988 ± 1.254	5.198 ± 5.636	1.912 ± 1.241	7.235 ± 6.279	2.431 ± 1.152	9.856 ± 4.769	3.031 ± 0.817
VP-Net	**1.585 ± 2.283**	**0.992 ± 0.775**	2.450 ± 2.903	1.274 ± 0.910	2.007 ± 3.502	**0.997 ± 1.007**	1.051 ± 1.681	0.863 ± 0.554

The results shown as mean ± standard deviation; The best values of MSE, and MAE for each human skin site and closed comedones highlighted in bold.

### 3.2 Influence of VP-Net size

To investigate the influence of VP-Net size on prediction performance and model efficiency, we varied the filter sizes utilized in VP-Net. Our proposed VP-Net architecture included five CBR blocks and three separable convolution blocks (Figure 2). The baseline VP-Net (VP-Net-B) was defined with initial filter sizes for the five CBR blocks set to $FS_{\text{CBR}}\in$ {16, 16, 32, 64, 128}, and for the three separable convolution blocks, $FS_{\text{SCB}}\in$ {32, 64, 128}. We also proposed two additional VP-Net sizes, called VP-Net-S ( $FS_{\text{CBR}}\in$ {16, 16, 16, 32, 64}; $FS_{\text{SCB}}\in$ {16, 32, 64}) and VP-Net-L ( $FS_{\text{CBR}}\in$ {32, 32, 64, 128, 256}; $FS_{\text{SCB}}\in$ {64, 128, 256}).

Table 4 and Table 5 compare the evaluation metrics among the three VP-Net sizes on agar phantoms and *in vivo* human skin datasets. VP-Net-L demonstrated relatively high performance in the 1%, 3%, 4.5% and 5% agar phantoms but did not achieve the best metrics for human skin. VP-Net-B achieved the lowest MSE (1.585) and MAE (0.992) on the back of hand, the lowest MAE (0.997) on the forearm, and similar MSE and MAE values to VP-Net-S on the palm. In the closed comedones, VP-Net-S had the best performance with MSE of 0.659 and MAE of 0.661.

**TABLE 4:** Comparison of VP-Net sizes for SAW velocity prediction on agar phantoms.

Model		Agar 1.0%	Agar 1.5%	Agar 2.0%	Agar 2.5%	Agar 3.0%	Agar 3.5%	Agar 4.0%	Agar 4.5%	Agar 5.0%
VP-Net-S	MSE	0.188 ± 0.085	0.121 ± 0.137	**0.197 ± 0.238**	**0.126 ± 0.182**	0.088 ± 0.096	2.770 ± 2.899	0.179 ± 0.223	0.717 ± 0.778	1.087 ± 1.079
VP-Net-B		1.158 ± 1.294	**0.121 ± 0.128**	0.369 ± 0.420	0.225 ± 0.256	0.057 ± 0.090	**0.742 ± 1.350**	**0.149 ± 0.192**	0.636 ± 0.696	0.995 ± 1.074
VP-Net-L		**0.178 ± 0.120**	0.130 ± 0.153	0.377 ± 0.416	0.204 ± 0.255	**0.042 ± 0.070**	4.268 ± 3.137	0.159 ± 0.188	**0.608 ± 0.684**	**0.884 ± 0.968**
VP-Net-S	MAE	0.420 ± 0.106	**0.291 ± 0.190**	**0.359 ± 0.261**	**0.275 ± 0.225**	0.250 ± 0.159	1.404 ± 0.894	0.346 ± 0.244	0.716 ± 0.452	0.892 ± 0.540
VP-Net-B		0.913 ± 0.569	0.295 ± 0.185	0.505 ± 0.338	0.393 ± 0.266	0.184 ± 0.150	**0.672 ± 0.539**	**0.313 ± 0.227**	0.673 ± 0.428	0.837 ± 0.542
VP-Net-L		**0.396 ± 0.144**	0.296 ± 0.205	0.512 ± 0.339	0.367 ± 0.264	0.157 ± 0.131	1.889 ± 0.835	0.329 ± 0.225	**0.653 ± 0.425**	**0.785 ± 0.517**

The results shown as mean ± standard deviation; The best values of MSE, and MAE for each agar phantom highlighted in bold.

**TABLE 5:** Comparison of VP-Net sizes for SAW velocity prediction on *in vivo* human skin.

Model	Back of hand		Palm		Forearm		Skin Face Acne	
	MSE	MAE	MSE	MAE	MSE	MAE	MSE	MAE
VP-Net-S	1.689 ± 2.093	1.065 ± 0.744	**2.329 ± 2.867**	**1.245 ± 0.883**	**2.002 ± 3.356**	1.035 ± 0.965	**0.659 ± 0.891**	**0.661 ± 0.471**
VP-Net-B	**1.585 ± 2.283**	**0.992 ± 0.775**	2.450 ± 2.903	1.274 ± 0.910	2.007 ± 3.502	**0.997 ± 1.007**	1.051 ± 1.681	0.863 ± 0.554
VP-Net-L	1.749 ± 2.391	1.058 ± 0.793	2.358 ± 2.721	1.252 ± 0.889	2.130 ± 3.478	1.059 ± 1.004	0.660 ± 0.599	0.722 ± 0.372

The results shown as mean ± standard deviation; The best values of MSE, and MAE for each human skin site and closed comedones highlighted in bold.

### 3.3 Model complexity analysis

**FIGURE 4:**
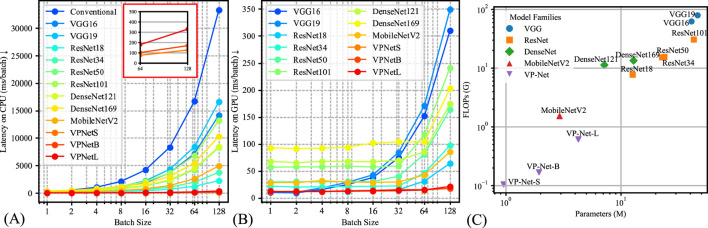
Model complexity comparison results. **(A)** Latency time of the various methods based on CPU (Intel i9-12900K). **(B)** Latency time of the various methods based on GPU (Nvidia RTX 4090). **(C)** Model parameters and floating-point operations (FLOPs) comparison.

The model’s inference efficiency among various batch sizes for input data was evaluated (Figure 4). We utilized the same computation platform to compare the processing time between the conventional velocity estimation method and the neural network-based methods, as shown in Figure 4A. Figure 4B demonstrates the inference time comparison between the neural networks. VP-Net performance outperformed the other neural networks on both CPU and GPU. Moreover, when the batch size increased, VP-Net achieved a higher throughput than the other networks. The model complexity comparison was compared based on the floating-point operations (FLOPs) and network parameters, as shown in Figure 4C. VP-Net family had the relatively lowest FLOPs compared to the other neural networks, and VP-Net-S and VP-Net-B have the lowest and second-lowest parameters, respectively.

### 3.4 Interpretation of proposed deep learning network

Gradient-weighted class activation maps (Grad-CAM) (Selvaraju et al., 2017) were employed to interpret the decision-making process of VP-Net when predicting wave velocity from a single raw phase slice. Distinct from the original Grad-CAM, which generated activation maps based on the model’s output class label, this experiment used the model-predicted SAW velocity to produce the Grad-CAMs. Based on the model architecture (Figure 2), we generated Grad-CAMs from the first convolution layer of each CBR block. These maps emphasize areas crucial for the model’s prediction, providing in-depth information on its internal operations.

**FIGURE 5:**
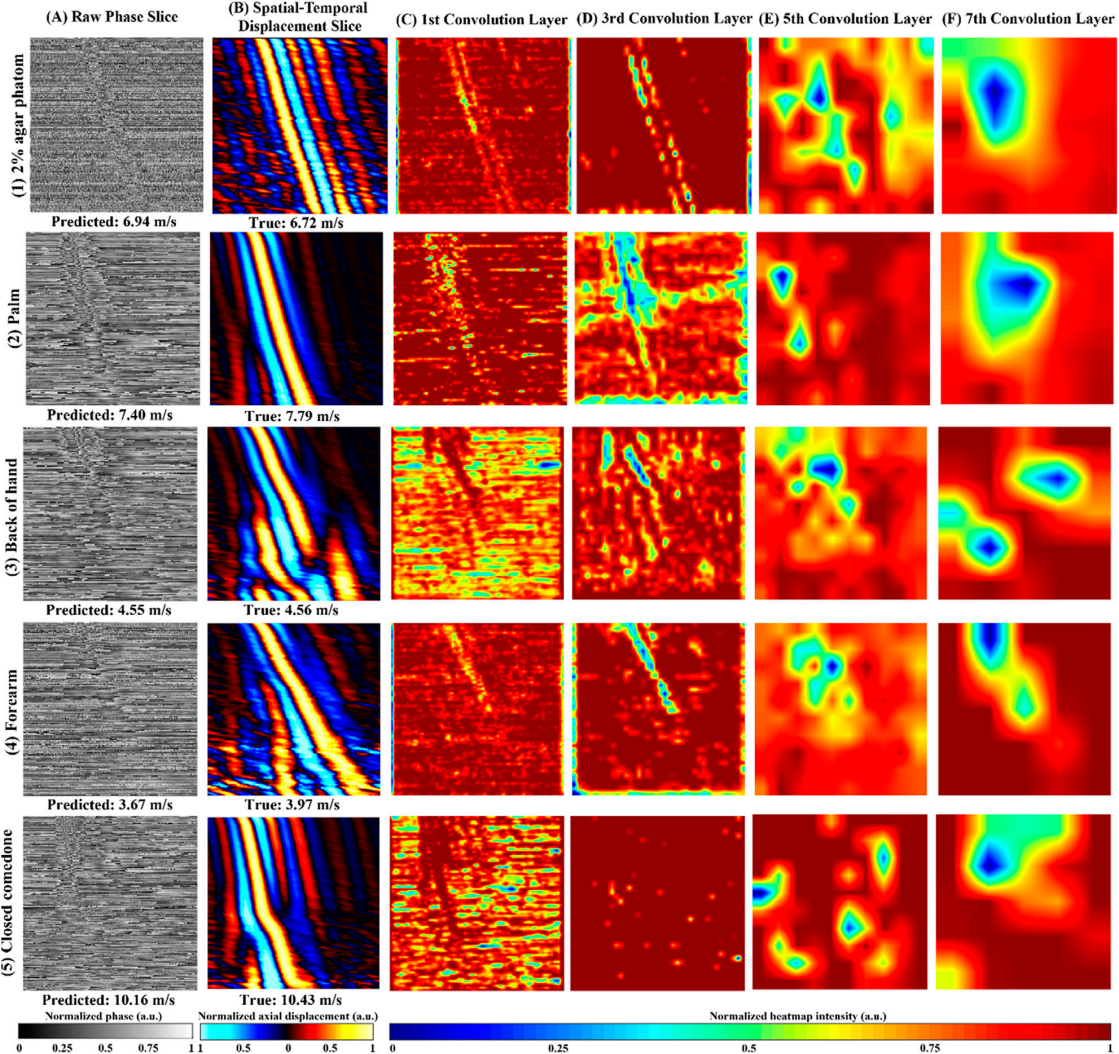
Repretative normalized raw phase slices, axial displacement slices, Gradient-weighted Class Activation Map (Grad-CAM) from the 2D convolution layers in VP-Net for tissue-mimicking phantom, *in vivo* human healthy skin site, and *in vivo* human abnormal skin. (1) 2% agar-based tissue-mimicking phantom, at 1,175 µm depth, (2) palm from a male in the 20s age group male, at 131.6 µm depth, (3) back of hand from a male in the 30s age group, at a depth of 197.4 µm, (4) forearm, a female in the 20s group, at a depth of 470 µm. (5) a closed comedo from a male in the 20s group, at a depth of 225.6 µm **(A)** raw phase slice, **(B)** axial displacement slice, **(C**–**F)** Grad-CAMs from the 1st, 3rd, 5th, and 7th 2D convolution layers, respectively; Predicted velocity by VP-Net and ground truth velocity for each sample displayed beneath raw phase and displacement slices, respectively.

Figure 5 shows an example of the raw phase slice from agar phantom (Figures 5, 1A), human healthy skin (Figures 5, 1–4A), and abnormal skin (Figures 5A), accompanied by their respective Grad-CAM (Figures 5C–F) produced from our purposed VP-Net. The raw phase slices were selected from the test set, which were not presented in the model training and validation stages. Corresponding axial displacement slices (Figure 5B) were used to estimate the ground truth SAWvelocity. Our VP-Net demonstrated high accuracy in velocity prediction for tissue-mimicking phantoms, three healthy skin sites, and abnormal skin, with differences between predicted and ground truth velocities being less than 0.3 m/s.

The perturbations caused by SAW propagation, surrounded by massive noise, were noticeable in all the raw phase slices (Figure 5A). Due to the homogeneous properties of the agar phantom, the processed displacement slice (Figures 5, 1B) displayed clear wave propagation and intense signals with less noise and fewer artifacts, even at a significant depth of 1,175 µm. The Grad-CAMs revealed a clear shape of the main wave propagation at the first and third convolution layers.

For the palm, which displayed a clear and distinct pattern of wave propagation on the displacement slice (Figures 5, 2B), the Grad-CAMs appeared to identify the main wave’s contour and texture, as reflected in the outputs of the first to third convolution layers (Figures 5, 2D, 1F). In the back of the hand, some distortion was observed, possibly caused by movement (Figures 5, 3B). Interestingly, the model seemed to recognize the main wave’s textures and shape, focusing less on the distorted region (Figures 5, 3C, 2G). The forearm slice, taken from a deeper depth (470 µm), exhibited more noise in its reconstructed displacement slice (Figures 5, 4B). Still, the model’s first to third convolution layers (Figures 5, 4C, 3D) appeared to capture the main wave’s texture.

Regarding the abnormal skin dataset, the wave pattern changed due to the boundary between closed comedones and the surrounding healthy skin at a lateral distance of 4.5 mm on the displacement slice (Figures 5B). In the first convolution layer (Figures 5C), only the SAW propagation across the closed comedo region was shown as a high-intensity pattern. From the deeper convolution layers of the agar phantom and human skins (Figures 5E, F), which likely indicated the high-level features extracted, intensity changes around the wave propagation region could be noticed.

### 3.5 Prediction of SAW velocities using VP-Net

The bulk SAW velocities of agar-based tissue-mimicking phantoms, *in vivo* healthy human skin, and abnormal skin were predicted using our trained VP-Net on the test set. The input raw phase slices from the test set were not included in the training and validation datasets. Table 6 summarizes the SAW velocities predicted by VP-Net, compared with the ground truth velocities estimated using the flight-of-flight approach.

**TABLE 6:** SAW velocities of agar-based tissue-mimicking phantoms, healthy skin at three sites between 20s and 30s age group, and abnormal skin estimated from time-of-flight approach and proposed VP-Net.

Sample	Number of OCE scans	Number of raw phase slices	Age group	Approach	SAW velocity (m/s)
1% agar	2	400	-	Time-of-flight	2.66 ± 0.09
				VP-Net	3.09 ± 0.05
1.5% agar	2	421	-	Time-of-flight	4.39 ± 0.32
				VP-Net	4.36 ± 0.13
2% agar	2	293	-	Time-of-flight	6.26 ± 0.32
				VP-Net	6.39 ± 0.28
2.5% agar	2	533	-	Time-of-flight	8.88 ± 0.25
				VP-Net	8.99 ± 0.22
3% agar	2	411	-	Time-of-flight	9.53 ± 0.18
				VP-Net	9.33 ± 0.12
3.5% agar	2	418	-	Time-of-flight	11.557 ± 0.49
				VP-Net	11.708 ± 0.52
4% agar	2	350	-	Time-of-flight	14.00 ± 0.34
				VP-Net	13.87 ± 0.22
4.5% agar	2	452	-	Time-of-flight	14.53 ± 0.42
				VP-Net	14.14 ± 0.41
5% agar	2	446	-	Time-of-flight	15.96 ± 0.51
				VP-Net	15.10 ± 0.29
Palm	8	1524	20s	Time-of-flight	6.72 ± 0.79
				VP-Net	6.78 ± 0.82
	9	1311	30s	Time-of-flight	8.32 ± 0.79
				VP-Net	8.10 ± 0.74
Forearm	6	1145	20s	Time-of-flight	4.35 ± 0.75
				VP-Net	4.29 ± 0.33
	7	865	30s	Time-of-flight	5.44 ± 1.24
				VP-Net	5.33 ± 1.06
Back of hand	6	616	20s	Time-of-flight	3.75 ± 0.34
				VP-Net	4.40 ± 0.29
	7	619	30s	Time-of-flight	4.31 ± 0.38
				VP-Net	4.55 ± 0.22
Closed comedones	3	642	20s	Time-of-flight	9.08 ± 0.69
				VP-Net	9.19 ± 0.41

The actual and predicted SAW velocities of the agar phantoms increased with concentration. The phantoms showed stability and consistency, showing that the mean predicted velocities were close to the actual velocities, indicated by a standard deviation of less than 0.5. For healthy human skin, the network-predicted bulk SAW velocities for both age groups (20s and 30s) across the three skin sites closely aligned with the actual velocities obtained from the conventional method. The palm exhibited the highest SAW velocities, approximately 8 m/s in the 30s group and 6 m/s in the 20s group, followed by the forearm, with approximately 4 m/s in the 20s group and 5 m/s in the 30s group. For the back of the hand, VP-Net predicted velocities were higher than those obtained by the conventional method by 0.6 m/s in the 20s group, and 0.2 m/s in the 30s group. For closed comedones, VP-Net predicted velocity was close to the conventional method, with a high velocity of approximately 9 m/s, indicating higher biomechanical properties.

## 4 Discussion

Wave-based OCE has been one of the most studied OCE branches, producing a fundamental impact in the quantitative and nondestructive biomechanical characterization of tissues. However, the long processing time limits its real-time and clinical applications (Sun et al., 2011). In this study, we proposed a rapid, high-efficiency, and high-accuracy deep-learning-based velocity prediction network (VP-Net) to predict biomechanical property-related velocity. We comprehensively evaluated the network with homogenous agar-based tissue-mimicking phantoms, *in vivo* human healthy and abnormal skin. Compared to the conventional OCE velocity estimation method (Zvietcovich and Larin, 2022), VP-Net could directly predict velocity from a single raw OCE slice, which provided end-to-end processing and eliminates the requirement for complex processing. Therefore, the proposed VP-Net has great potential to be translated into clinical practice for characterizing skin aging, as well as assessing and managing the treatment of acne vulgaris.

In the discussion, the results will be analyzed and compared with the findings from other studies. First, we conducted a comprehensive comparison with a series of existing deep-learning models, including VGG16/19, ResNet18/34/50/101, DenseNet121/169, and MobileNetV2. The evaluation results in Table 1 and Table 2 show that the mean MSE and MAE errors were approximately below 0.5 in agar phantoms, with concentrations ranging from 1.5% to 4%, indicating high accuracy in predicting the velocities in these agar phantoms. However, for the agar phantoms with low (1%) and higher concentrations (4.5% and 5%), the mean errors from VP-Net were relatively higher than 0.5. We hypothesize this is due to the unbalanced data distribution in the training datasets, as the velocity distributions for these concentrations had fewer slices (5,127 slices). Regarding the *in vivo* human skin (Table 3), VP-Net achieved the best performance in the back of hand (MSE: 1.585; MAE: 0.992) and had the lowest MAE of 0.863 in the forearm. In the palm, VP-Net performed similarly to ResNet101 in terms of MSE and MAE. For closed comedones, VP-Net had the second-lowest MSE and MAE. Thus, VP-Net demonstrated high accuracy in predicting biomechanical property-related velocities, indicating its potential for early diagnosis of skin conditions.

An ablation study was conducted to investigate the influence of VP-Net sizes on performance. As shown in Table 4, increasing the size of VP-Net did not improve accuracy for agar phantoms with 1.5%–4.0% concentrations. However, for agar phantoms with 1.5%–2.5% concentrations, decreasing the size of VP-Net improved performance. In the human skin dataset (Table 5), VP-Net-B provided the lowest MAE (0.992) and MSE (1.585) errors in the back of hand, and the lowest MAE (0.997) and second-lowest MSE (2.007) in the forearm. In the palm and closed comedones, reducing the size of VP-Net again provided the best performance in terms of MSE and MAE errors. Compared to VP-Net-S and VP-Net-L, VP-Net-B offered the best trade-off between prediction performance and model complexity.

Additionally, we evaluated the computational demand of VP-Net in both GPU and CPU environments, comparing inference time and model complexity among various methods, as presented in Figure 4. Figure 4A, B illustrate that VP-Net had the lowest inference time in both environments. Specifically, Figure 4A shows that VP-Net accelerated the velocity prediction procedure by a factor of 100 compared to the conventional method. Figure 4B further indicates that VP-Net-S and VP-Net-B had the lowest model complexity and network parameters, respectively.

Grad-CAM (Figure 5) was employed to interpret VP-Net’s velocity prediction processes. When the wave propagation pattern was clear and had single wave mode details (Figure 5 (1B and 2B)), the full wave propagation path (Figure 5 (1C,D and 2 C,D)), was seen in the shallow convolution layers (first to third). In contrast, when artifacts induced by motion, far-end noise, or low intensities at deeper depths were present (Figures 5, 3B, 4B), only the high-quality portions of the wave patterns were emphasized in these layers (Figures 5, 3C, D). This may indicate that the model effectively filtered significant noise from the raw phase slices to extract useful and accurate wave information. For abnormal skin, only the high-velocity wave propagating through the comedo region was displayed as the highest intensity curve in the first convolution layer (Figure 5C). We believe that the comprehensive training dataset, which included high-quality slices at surface depths, low-intensity wave images at deeper depths, motion artifacts, and boundaries between abnormal and healthy regions, enhanced the model’s ability to analyze difficult situations and accurately predict the velocity of abnormal regions (Li et al., 2022).

Biomechanical properties, specifically elasticity (Young’s modulus), can be estimated directly from velocity measurements. The bulk Young’s modulus ( E ) can be calculated from the predicted SAW velocity ( $\hat{V_{R}}$ ) using the formula $E=3.35\times {\hat{V_{R}}}^{2}$ , assuming skin mass density (ρ) of 1.02 g/cm^2^ and a Poisson’s ratio (υ) of 0.5 (Liang and Boppart, 2009). By converting the velocities in Table 6 to elasticity, the bulk Young’s modulus for 1%–2% agar phantoms predicted from raw phase slices were 44 ± 14 kPa, 66 ± 4 kPa, and 149 ± 14 kPa, respectively. These results are in good agreement with the values obtained by Yang et al. (Yang et al., 2022). The Young’s modulus for the 2% agar phantom predicted by our model was 286 ± 12 kPa, comparable to the 254 kPa reported by Brewin et al. (Brewin et al., 2015). In our study, the average predicted Young’s moduli for the three skin sites were 188 ± 58 kPa for the palm, 79 ± 42 kPa for the forearm, and 64 ± 35 kPa for the back of hand, was consistent with values documented in our previous study (Zhou et al., 2020). Also, the values aligned with other reference values. For instance, The bulk Young’s modulus for the palm was reported at 108 ± 48 kPa (Zhang et al., 2011), forearm at 42 ± 32 kPa (Zhang et al., 2011) and 129 ± 88 kPa (Diridollou et al., 2000); Back of hand 11–23 kPa (Wakhlu et al., 2017). Notably, a difference in bulk Young’s modulus between age groups (20s and 30s) was observed: for the palm, 154 ± 37 kPa in the 20s group vs 220 ± 40 kPa in the 30s group; for the forearm, 62 ± 9 kPa in the 20s group vs 95 ± 38 kPa in the 30s group; and for the back of the hand, 65 ± 9 kPa in the 20s group vs 69 ± 7 kPa in the 30s group. Regarding abnormal skin, no previous studies have reported the Young’s modulus of facial skin diseases. For comparison, the mean stiffness of malignant neck tumors was 226.4 kPa as measured by ultrasound elastography (Roldán, 2016), similar to the predicted Young’s modulus of closed comedones at 308 ± 35 kPa. Thus, our proposed VP-Net demonstrated its efficacy by accurately obtaining bulk velocity from a single image with noisy raw phase information.

Neidhardt et al. (2021) reported a densely connected network for predicting concentrations of gelatin phantoms by analyzing shear wave OCE data. They later expanded this approach to aid in force estimation on gelatin phantoms and *ex vivo* chicken hearts (Neidhardt et al., 2023). Their model could process both 3D (depth × lateral distance × time) and 4D (depth × lateral distance × vertical distance × time) volumes, with each dimension of 32 pixels, and was capable of performing classification in real-time. While their methods had valuable contributions, particularly for real-time and 4D analysis, there may be challenges when applying this approach to *in vivo* studies and clinical translations. First, the input depth for each volume in their model required 32 pixels, approximately 235 µm. In contrast, our proposed deep learning network could predict velocity from each single slice, with a single depth layer of approximately 4.7 µm. In addition, their low spatial sampling points limited the spatial resolution of the raw volume, resulting in reduced elastography resolution (Kirby et al., 2019). This constrains its applications to address motion artifacts and complicated wave patterns, which frequently occur *in vivo* OCE acquisitions. Next, its field of view was restricted to 3 mm, which could be insufficient for measuring abnormal skin conditions, typically around 6 mm in diameter (Kasmi and Mokrani, 2016). In our study, the scanning range was up to 11 mm, and we successfully predicted the bulk velocities of closed comedones with diameters greater than 4.2 mm. Thus, VP-Net may offer an advantage in predicting biomechanical property-related velocity from a single image, handling high noise and artifacts, and is particularly suitable for both healthy and abnormal *in vivo* scans.

While our work represents a significant advancement, further research is needed to refine the deep learning model, particularly its translation to clinical settings. By including a more diverse range of participants, we intend to enhance the robustness of our model, ensuring accurate wave velocity predictions across all biological genders. Additionally, we plan to substantially enlarge our dataset to explore the potential of vision transformers for predicting the biomechanical properties of both healthy and abnormal human skin.

## 5 Conclusion

In conclusion, we developed an end-to-end deep learning-based velocity prediction network (VP-Net) for predicting elastic wave velocities associated with biomechanical properties using OCE. VP-Net demonstrated the ability to provide real-time elastic wave velocity predictions without the need for expertise and complex image processing. In *vivo* applications on both healthy and abnormal human skin, VP-Net accurately differentiated age-related changes in elastic velocities across multiple skin sites and detected high velocities in closed comedones. Therefore, VP-Net holds significant potential for clinical applications in characterizing skin aging, as well as assessing and managing the treatment of acne vulgaris.

## Data Availability

The raw data supporting the conclusions of this article will be made available by the authors, without undue reservation.
